# TWEAK promotes exercise intolerance by decreasing skeletal muscle oxidative phosphorylation capacity

**DOI:** 10.1186/2044-5040-3-18

**Published:** 2013-07-08

**Authors:** Shuichi Sato, Yuji Ogura, Vivek Mishra, Jonghyun Shin, Shephali Bhatnagar, Bradford G Hill, Ashok Kumar

**Affiliations:** 1 Department of Anatomical Sciences and Neurobiology, University of Louisville School of Medicine, 500 South Preston Street, Louisville, KY 40202, USA; 2 Diabetes and Obesity Center, Institute of Molecular Cardiology, and Department of Medicine, University of Louisville, Louisville, KY 40202, USA; 3 Present address: Gastroenterology Division, University of Pittsburgh School of Medicine, Pittsburgh, PA 15261, USA

**Keywords:** Skeletal muscle, Exercise tolerance, TWEAK, Fn14, PGC-1α, PPARδ

## Abstract

**Background:**

Proinflammatory cytokine tumor necrosis factor (TNF)-like weak inducer of apoptosis (TWEAK) and its receptor Fn14 are the major regulators of skeletal muscle mass in many catabolic conditions. However, their role in muscle metabolism remains largely unknown. In the present study, we investigated the role of TWEAK on exercise capacity and skeletal muscle mitochondrial content and oxidative metabolism.

**Methods:**

We employed wild-type and TWEAK-knockout (KO) mice and primary myotube cultures and performed biochemical, bioenergetics, and morphometric assays to evaluate the effects of TWEAK on exercise tolerance and muscle mitochondrial function and angiogenesis.

**Results:**

TWEAK-KO mice showed improved exercise tolerance compared to wild-type mice. Electron microscopy analysis showed that the abundance of subsarcolemmal and intermyofibrillar mitochondria is significantly increased in skeletal muscle of TWEAK-KO mice compared to wild-type mice. Furthermore, age-related loss in skeletal muscle oxidative capacity was rescued in TWEAK-KO mice. Expression of a key transcriptional regulator peroxisome proliferator-activated receptor γ coactivator 1α (PGC-1α) and several other molecules involved in oxidative metabolism were significantly higher in skeletal muscle of TWEAK-KO mice. Moreover, treatment of primary myotubes with soluble TWEAK inhibited the expression of PGC-1α and mitochondrial genes and decreased mitochondrial respiratory capacity. Deletion of TWEAK also improved angiogenesis and transcript levels of vascular endothelial growth factor in skeletal muscle of mice.

**Conclusions:**

These results demonstrate that TWEAK decreases mitochondrial content and oxidative phosphorylation and inhibits angiogenesis in skeletal muscle. Neutralization of TWEAK is a potential approach for improving exercise capacity and oxidative metabolism in skeletal muscle.

## Background

Skeletal muscle is the largest tissue of the human body which ensures basic functions such as locomotion, metabolism, and respiration. Skeletal muscle exhibits a high level of plasticity in response to physiological stressors. For example, in response to exercise training, the proportion of slow-type fibers and mitochondrial content within fibers is noticeably increased [1]. The biogenesis of new mitochondria and clearance of defunct mitochondria are essential to meet cellular energy demand especially during endurance exercise and to protect from many chronic conditions such as diabetes, heart failure, obesity, and aging [2,3]. Peroxisome proliferator-activated receptor (PPAR)-γ coactivator 1α (PGC-1α) is a key player in regulating skeletal muscle fiber composition, mitochondrial content, and oxidative metabolism, and maintenance of glucose, lipid, and energy homeostasis in response to physiological demands [4-6]. Transgenic mice overexpressing physiological levels of PGC-1α in skeletal muscle have increased mitochondrial biogenesis and oxidative capacity, are more resistant to fatigue and have improved aerobic performance [7-10]. Forced expression of PGC-1α also preserves skeletal muscle mass in various catabolic states including denervation [11,12]. PGC-1α improves mitochondrial biogenesis by coactivating nuclear respiratory factor (NRF)-1 [13], NRF-2 [14] and estrogen-related receptor α [15-17] which, in turn, augments the expression of hundreds of nuclear-coded mitochondrial genes [18]. Recent evidence also suggests a role for mitochondria in preventing muscle protein degradation in catabolic conditions [19,20]. Thus, preserving mitochondria content and function is essential to countering muscle wasting and to improving muscle homeostasis.

Inflammatory cytokines such as tumor necrosis factor-α (TNF-α) and interleukin-6 (IL-6) are some of the important mediators of loss of skeletal muscle mass and function in many catabolic conditions [21-24]. TNF-α has been reported to augment muscle protein degradation both *in vitro* and *in vivo*[25-27]. Moreover, TNF receptor I knockout (KO) mice are protected from diet-induced obesity due to increased thermogenesis [28]. Importantly, TNF-α inhibits the expression of PGC-1α levels in myotubes [29]. In animal models of cancer cachexia, mitochondrial content and biogenesis are reduced with increased levels of IL-6 during the progression of the condition whereas inhibition of IL-6 activity attenuates tumor-induced loss of skeletal muscle [30,31]. It is also notable that while moderate exercise attenuates inflammation [32-34], acute exercise more robustly increases the levels of inflammatory cytokines in obese compared with lean subjects suggesting that rigorous exercise can further aggravate the condition of overweight individuals [35]. Despite these observations, cause-and-effect relationships between different inflammatory mediators, exercise tolerance, and mitochondrial function in skeletal muscle have not been clearly established using genetic mouse models.

TNF-like weak inducer of apoptosis (TWEAK) is a major proinflammatory cytokine of the TNF super family [36] that functions by binding to Fn14 receptors on target cells [37-39]. TWEAK-Fn14 signaling mediates unique and context-dependent pleiotropic effects [40]. TWEAK has been recently identified to be a key mediator that causes skeletal muscle loss that occurs in response to denervation, immobilization, and starvation [21,41,42]. Moreover, TWEAK-transgenic mice overexpressing physiological levels of TWEAK in skeletal muscle showed a higher proportion of fast-type fibers (glycolytic) in soleus and extensor digitorum longus (EDL) muscle [41] indicating that elevated levels of TWEAK cause a slow-to-fast type fiber switch. However, the role and mechanisms by which TWEAK affects exercise capacity and bioenergetic function have not been studied.

In the present study, we have used TWEAK-knockout (KO) mice to investigate the role of TWEAK in exercise tolerance and skeletal muscle bioenergetic function. Our results show that, compared with wild-type mice, TWEAK-KO mice have increased exercise tolerance, which is associated with higher levels of skeletal muscle subsarcolemmal and intermyofibrillar mitochondria and enhanced oxidative phosphorylation capacity. TWEAK represses the expression of PGC-1α and several other molecules involved in mitochondrial biogenesis and oxidative metabolism *in vivo* and in cultured myotubes. Furthermore, vascularization and expression of vascular endothelial growth factor (VEGF) are increased in skeletal muscle of TWEAK-KO mice compared with wild-type mice. These results support our hypothesis that TWEAK causes exercise intolerance by suppressing mitochondrial oxidative metabolism and angiogenesis.

## Methods

### Cell culture

Primary myoblasts were isolated from hind limb muscle of mice and cultured following the same protocol as previously described [43]. Briefly, hind limb skeletal muscles from mice were aseptically isolated, minced into a coarse slurry, and enzymatically digested at 37°C for one hour by adding 200 IU/ml collagenase I (cat # LS004196; Worthington, Lakewood, NJ, USA) and 0.1% pronase (EMD Chemicals, Billerica, MA, USA). The digested slurry was filtered through a 70 μm filter and spun, and isolated cells were resuspended and cultured initially in F-10 medium (containing 20% fetal bovine serum (FBS) and supplemented with 10 ng/ml basic fibroblast growth factor) and then in F-10 plus (Dulbecco’s) Modified Eagle’s Medium ((D)MEM) (1:1 ratio) based culture medium supplemented with 15% FBS on culture dishes coated with 10% matrigel (BD Biosciences, San Jose, CA, USA). Differentiation in primary myoblast cultures was induced by replacing the growth medium with differentiation medium (2% horse serum in (D)MEM).

### Animals

TWEAK-KO mice were provided by Dr. Avi Ashkenazi (Genentech South, San Francisco, CA, USA) and have been previously described [40]. All the mice were in the C57BL/6 background, and their genotype was determined by PCR from tail DNA. All animal procedures were approved (protocol # 10129) by the Institutional Animal Care and Use Committee and conformed to the American Physiological Society’s Guiding Principles in the Care and Use of Animals.

### Treadmill running protocol

Mice were subjected to treadmill running following the same previously described protocol [44]. In brief, 4.5-month-old wild-type and TWEAK-KO mice were matched for body weight and randomly assigned to either a sedentary or exercise group. Mice were subjected to an acute bout of treadmill (Eco3/6 treadmill; Columbus Instruments, Columbus, OH, USA) running at 15 m/minute for 90 minutes. All mice in the exercise group finished the 90-minute trial and were visibly exhausted. Mice were sacrificed within 30 minutes after exercise to study mitochondrial function.

### Exercise tolerance test

The exercise tolerance test on mice was performed following a method as previously described [45]. Briefly, all animals were run on a treadmill (Columbus Instruments) at 10 m/minutes for five minutes at 0% degree incline for acclimation for three days. On the exercise testing day, animals ran on the treadmill with a fixed slope of 10%. Mice first ran at 10 m/minute for five minutes and the speed was increased by 2 m/minute every two minutes until they were exhausted or a maximal speed of 46 m/minute was achieved. The criterion of exhaustion was defined as the inability of the animal to run on the treadmill for 10 seconds despite mechanical prodding. Running time and maximum speed achieved was measured whereas running distance, work and power were calculated.

### Transmission electron microscopy

Soleus muscles from wild-type and TWEAK-KO mice were fixed in 3% glutaraldehyde in 0.1 M cocodylate buffer overnight followed by fixing in 1% cocodylate-buffered osmium tetroxide. The tissue was dehydrated through a series of graded alcohols, and embedded in LX-112 plastic (Ladd Research Industries, Williston, VT, USA). Longitudinal sections (80 nm) were cut using an ultramicrotome (LKB, Rockville, MD, USA)) and stained with uranium acetate and lead citrate. Samples were analyzed using a transmission electron microscope (Philips CM 12; HZB, Berlin, Germany) operating at 60 kV. The pictures were captured at 8,800x magnification using a 3.2 megapixel digital camera (Sia-7C; Kodak, Rochester, NY, USA) at room temperature. No imaging medium was used to visualize the pictures, and images were stored as JPEG files. Image levels were equally adjusted using Photoshop CS2 software.

### Succinate dehydrogenase (SDH) staining

SDH staining was performed as previously described [46]. Briefly, transverse sections (8 μm) were cut from the mid-belly of the TA muscles on a cryostat at −20°C and stored at −80°C until SDH staining was performed. The sections were dried at room temperature for 30 minutes before incubation in a solution made up of 0.2 M phosphate buffer (pH 7.4), 0.1 M MgCl_2_, 0.2 M succinic acid (Sigma Chemical Company, St. Louis, MO, USA) and 2.4 mM nitroblue tetrazolium (NBT, Sigma) at 37°C in a humidity chamber for 45 minutes. The sections were then washed in deionized water for three minutes, dehydrated in 50% ethanol for two minutes, and mounted for viewing with DPX mount medium (Electron Microscopy Sciences, Hatfield, PA, USA). Digital photographs were taken from each section at 10X magnification under a Nikon Eclipse TE 2000-U microscope (Nikon, Melville, NY, USA) with a Nikon digital camera (Digital Sight DS-Fi1), and fibers were quantified with imaging software (Image J, NIH). At least 700 fibers were counted to determine SDH-positive fibers in each section in a blinded fashion. The percentage of SDH stained fibers was then determined based on a criteria using integrated optical density.

### Measurement of mitochondrial bioenergetics

Mitochondrial oxidative capacity was measured in isolated mitochondria and in cultured myotubes using a Seahorse Bioscience XF24 extracellular flux analyzer (Billerica, MA, USA). For measurements in isolated mitochondria, tissue from the limb muscle (approximately 50 mg) was isolated within 30 minutes of exercise and homogenized in 1 ml of isolation buffer (220 mM mannitol, 70 mM sucrose, 5 mM 3-(N-morpholino)propanesulfonic acid (MOPS), 1 mM ethylene glycol tetraacetic acid (EGTA), 0.3% fatty acid-free BSA, pH 7.2) using a Potter Elvehjem tube and a Teflon pestle. The homogenate was centrifuged at 500×g for five minutes at 4°C. The supernatant containing mitochondria was centrifuged at 10,000 × g for five minutes. After two wash-centrifugation steps in BSA-free isolation buffer, the mitochondria were suspended in respiration buffer (120 mM KCl, 25 mM sucrose, 10 mM 4-(2-hydroxyethyl)-1-piperazineethanesulfonic acid (HEPES), 1 mM MgCl_2_, 5 mM KH_2_PO_4_, pH to 7.2). Protein in the mitochondrial suspension was estimated using the Lowry DC assay (Biorad, Hercules, CA, USA) and 5 to 12.5 μg of mitochondrial protein was sedimented in XF culture plates as described previously [47]. Complex I-mediated, state 3 respiratory activity was determined by measuring the oxygen consumption rate (OCR) after injection of pyruvate (5 mM), malate (2.5 mM) and ADP (1 mM). The OCR of mitochondria after exposure to oligomycin (1 μg/ml) was used to estimate state 4 activity; exposure to carbonyl cyanide *p*-trifluoromethoxyphenylhydrazone (FCCP, 2 μM) was used to examine the uncoupled rate of respiration. Finally, succinate (10 mM) and rotenone (1 μM) were injected to assess maximal Complex II-mediated respiratory capacity. Data are expressed as pmol O_2_/min/μg protein. For measurements in cultured cells, differentiated myoblasts were exposed to soluble TWEAK (R&D Systems, Minneapolis, MN, USA) for 72 hours followed by the mitochondrial function assay outlined previously [48-51].

### RNA isolation and quantitative real time-PCR

RNA isolation and quantitative real time-PCR (qRT-PCR) were performed as previously described [41]. Briefly, total RNA was isolated from homogenized mouse tissues using the TRIzol reagent (Invitrogen, Grand Island, NY, USA) and an RNeasy Mini kit (QIAGEN, Valencia, CA, USA) according to the manufacturers’ protocols. First strand cDNA for PCR analyses were made using a reverse transcription system with 1 μg of purified RNA using oligo (dT) primer (Applied Biosystems, Grand Island, NY, USA) and the Omniscript reverse transcription kit (QIAGEN). The quantification of mRNA expression was performed using the SYBR green dye method on a sequence detection system (Applied Biosystems, model 7300). Primers were designed using Vector NTI software (Invitrogen). Primer sequences are available on request. The thermal conditions consisted of an initial denaturation at 95°C for 10 minutes, followed by 40 cycles of denaturation at 95°C for 15 seconds, annealing and extension at 60°C for one minute, and, for a final step, a melting curve of 95°C for 15 seconds, 60°C for 15 seconds, and 95°C for 15 seconds. All reactions were performed in duplicate to reduce variation. Data normalization was accomplished using the endogenous control (β-actin), and the normalized values were subjected to a 2^-ΔΔCt^ formula to calculate the fold change between control and experimental groups.

### Immunostaining for CD31

After cutting 8 μm thickness frozen sections of TA muscles, the sections were fixed by cold acetone for 10 minutes and dried in air for 30 minutes. The tissues were rinsed with phosphate buffered saline (PBS) twice, blocked in 2% BSA solution for one hour at room temperature followed by incubation with primary antibodies in 2% BSA solution (laminin, anti-rabbit 1:300, Sigma and CD31, anti-mouse 1:30, BD Biosciences, San Jose, CA, USA) at 4°C in a humidity chamber overnight. The next day, the sections were rinsed with PBS for five minutes three times and incubated with secondary antibody (Alexa Fluor-conjugated 488 anti-rabbit, 1:2500 and 546 anti-mouse, 1:2500, Invitrogen) in 2% BSA solution for one hour at room temperature. After washing twice with PBS and once with deionized water, the sections were mounted with DPX mounting medium. Digital photographs were taken from each section under a Nikon Eclipse TE 2000-U microscope (Nikon) with a Nikon digital camera (Digital Sight DS-Fi1). Number of CD31-postive vessels per myofiber was quantified using NIS Elements BR 3.00 software (Nikon).

### Statistical analysis

The results are presented as means ± standard deviation (SD). Student’s *t*-test was used to compare the difference between control and treatment groups. A value *P* <0.05 was considered statistically significant.

## Results

### Improvement in exercise tolerance in TWEAK-KO mice

**Figure 1:**
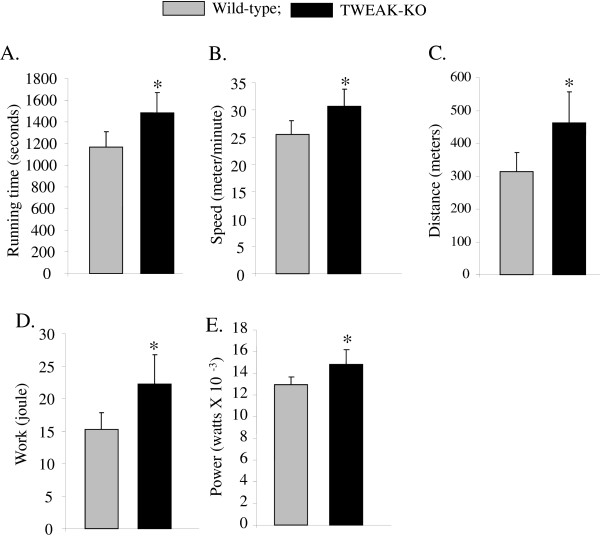
**Treadmill exercise tolerance test.** After acclimatization, mice were run on a treadmill with a 10% slope and increasing speed to exhaustion. Maximum speed and running time were monitored, and distance, work, and power were calculated based on the individual performance. **(A)** Running time; **(B)** Speed; **(C)** Distance; **(D)** Work; and **(E)** Power. Data are represented as mean ± SD. N = 6 in each group. *P <0.05; values vary significantly between wild-type and TWEAK-KO mice. KO, knockout.

We first studied the role of TWEAK in exercise tolerance in mice. Male 4.5-month old wild-type and TWEAK-KO mice were acclimated for three days for treadmill running prior to the exercise tolerance test. After acclimation, the mice were run on a 10% slope until the mice were unable to run on the treadmill for 10 seconds despite mechanical prodding. TWEAK-KO mice ran longer compared to wild-type mice (1,483 seconds for TWEAK-KOs versus 1,170 seconds for the controls, Figure 1A). Since TWEAK-KO mice could keep running on higher speed (30.6 m/minute for TWEAK-KOs versus 25.5 m/minute for the controls, Figure 1B), the difference in running distance became greater than 47% (463 meter for TWEAK-KOs versus 314 meter for the controls, Figure 1C). Work and power generated by TWEAK-KO mice were also higher compared to wild-type mice. TWEAK-KO mice generated 22.2 J of work while control mice generated 15.2 J of work (a difference of 46%) during the exercise test (Figure 1D). In turn, TWEAK-KO mice exerted 14% more power than the controls (14.9 mW for TWEAK-KO versus 13.0 mW for the wild-type, Figure 1E) on the treadmill. These data established that TWEAK-KO mice have significantly improved exercise tolerance.

### Ablation of TWEAK improves mitochondrial content in skeletal muscle of mice

**Figure 2:**
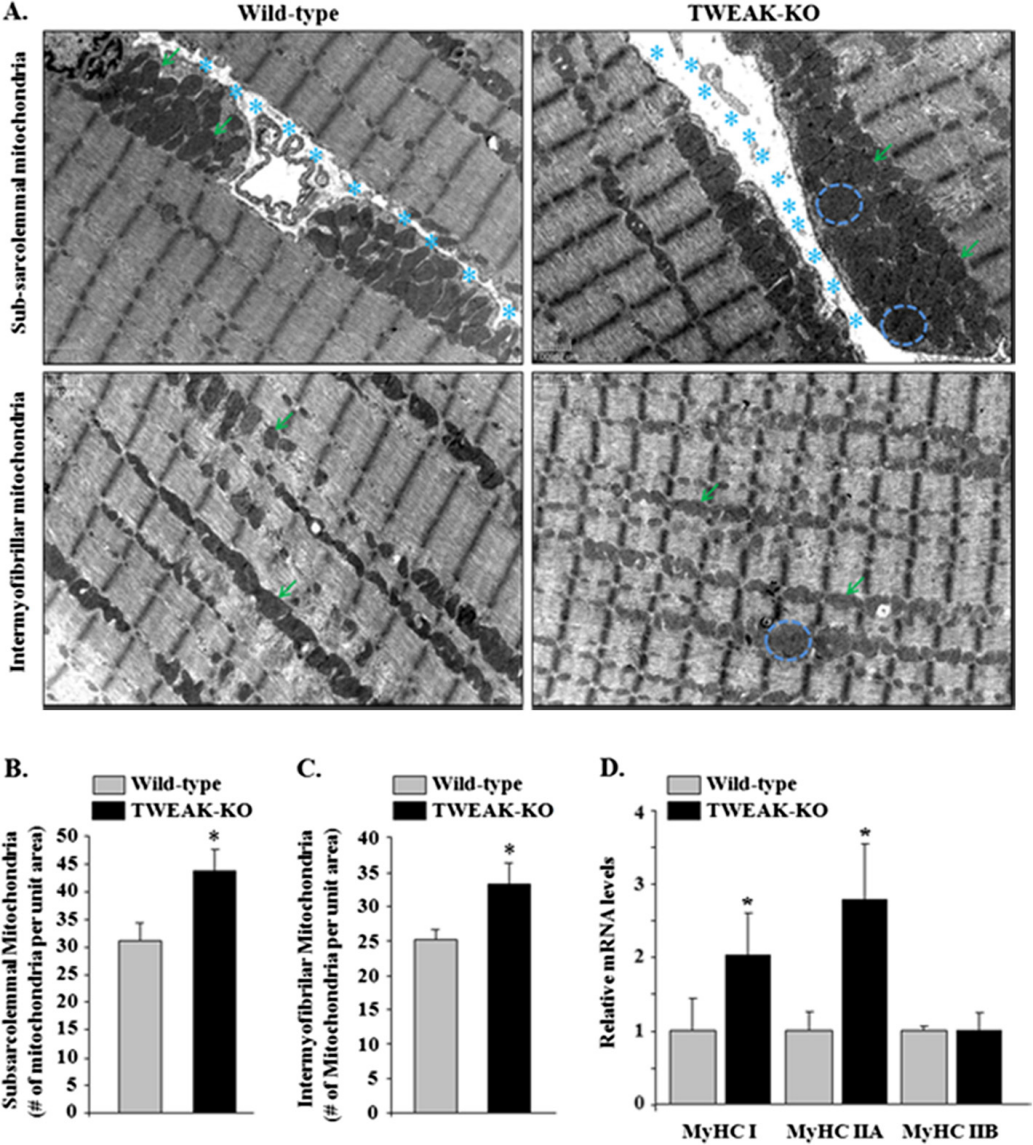
**Transmission electron microscopy (TEM) analysis for mitochondria morphology and content and expression of muscle fiber type-specific genes in TWEAK-KO mice.** Soleus muscle of six-month-old wild-type and TWEAK-KO mice were isolated and longitudinal sections were processed for TEM analysis. **(A)** Representative images of longitudinal soleus muscle are shown. The abundance and the size of mitochondria were increased in TWEAK-KO mice compared to wild-type mice. Arrows point to mitochondria in muscle sections. Circles are used to show representative enlarged mitochondria. Stars (‘*’) point to subsarcolemmal space in longitudinal sections. Scale bar: 1 μm. **(B)** Quantification of the number of subsarcolemmal mitochondria in soleus muscle of wild-type and TWEAK-KO mice. **(C)** Quantification of the number of intermyofibrillar mitochondria in soleus muscle sections of wild-type and TWEAK-KO mice. **(D)** Relative mRNA levels of MyHC type I, IIA and IIB in gastrocnemius muscle of 4.5-month-old wild-type and TWEAK-KO mice. Data are represented as mean ± SD. N = 3 in each group. *P <0.05; values vary significantly from wild-type mice. KO, knockout; TWEAK, TNF-like weak inducer of apoptosis.

We have previously shown that deletion of TWEAK increases the proportion of type I fibers in soleus and EDL muscles of mice [41]. In this study, we investigated whether TWEAK affects mitochondrial content in skeletal muscle of mice. The soleus muscle of 4.5-month old TWEAK-KO and wild-type mice were isolated and used to measure mitochondrial content by performing transmission electron microscopy. As shown in Figure 2A, the abundance and size of mitochondria was found to be increased in TWEAK-KO compared to wild-type mice. Quantitative analysis of mitochondria in electron micrographs showed that the levels of subsarcolemmal and intermyofibrillar mitochondria were increased by 42% and 32%, respectively, in TWEAK-KO mice compared to wild-type mice (Figure 2B and Figure 2C). Because gastrocnemius (GA) muscle contains a mixture of slow and fast twitch fibers and greatly influences running capacity on the treadmill, we measured mRNA levels of fiber type-specific myosin heavy chain (MyHC) isoforms in GA muscle of wild-type and TWEAK-KO mice. As shown in Figure 2D, mRNA levels of MyHC I and MyHC IIA were increased by 2- and 2.7-fold, respectively in TWEAK-KO mice compared to wild-type mice. These results further suggest that genetic ablation of TWEAK increases the slow-type fiber phenotype in skeletal muscle of mice.

**Figure 3:**
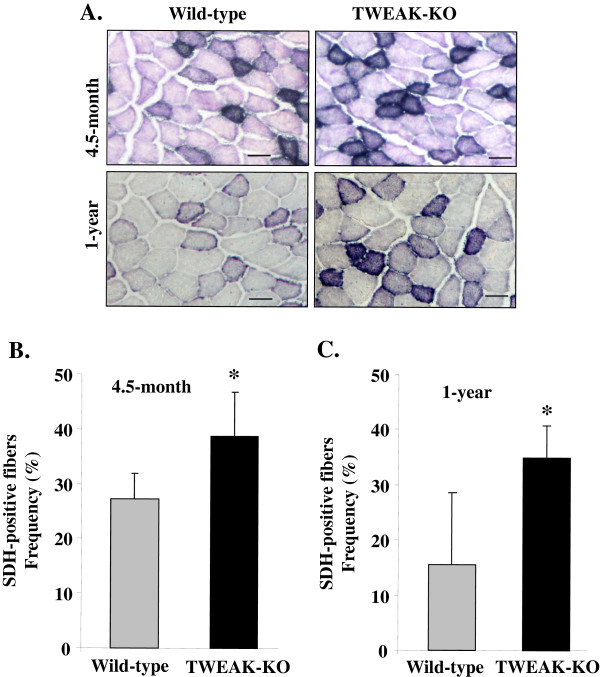
**Succinate dehydrogenase (SDH) staining analysis for oxidative capacity in TA muscle of TWEAK-KO mice.** Frozen transverse TA muscle sections from 4.5- or 12-month old wild-type and TWEAK-KO mice were used to stain for SDH. **(A)** Representative SDH-stained images are presented here. TA muscle of TWEAK-KO showed relatively dark staining for SDH compared to wild-type mice. Scale bars: 50 μm. Quantification of SDH-positive fibers in TA muscle of **(B)** 4.5-month, and **(C)** 12-month old wild-type and TWEAK-KO mice. Data are presented as mean ± SD. *P <0.05; values vary significantly from wild-type mice. KO, knockout; TA, tibial anterior; TWEAK, TNF-like weak inducer of apoptosis.

SDH, also known as complex II in the mitochondrial respiratory chain, is a marker of oxidative capacity of skeletal muscle at the fiber level. Therefore, we performed SDH staining on TA muscle of 4.5-month- and 12-month-old wild-type mice and TWEAK-KO mice and quantified the percentage of SDH-positive fibers. Compared to wild-type mice, fibers in TWEAK-KO mice stained more darkly for SDH (Figure 3A). Furthermore, there was a significant increase in the number of SDH-positive fibers in TWEAK-KO mice compared to wild-type mice at the age of 4.5 months (Figure 3B). Likewise, 12-month-old TWEAK-KO had more SDH-positive fibers compared to age-matched wild-type mice (Figure 3A and Figure 3C). Taken together, these results suggest that ablation of TWEAK in mice is sufficient to improve mitochondrial content and oxidative capacity of skeletal muscle.

### TWEAK-KO mice demonstrate increased expression of PGC-1α and metabolic genes in skeletal muscle

**Figure 4:**
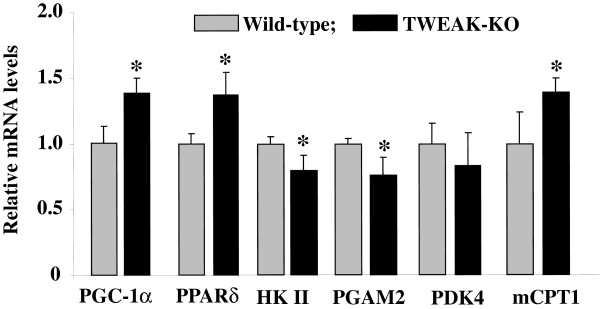
**qRT-PCR analysis of transcript levels of metabolic genes in skeletal muscle of wild-type and TWEAK-KO mice.** TA muscle from 4.5-month-old wild-type and TWEAK-KO mice were isolated and processed to measure mRNA levels of PGC-1α, PPARδ, HK II, PGAM2, PDK4, and mCPT1. Data are represented as mean ± SD. N = 4 in each group. *P <0.05. values vary significantly from wild-type mice. HK II, hexokinase-2; KO, knockout; mCPT1, mitochondrial carnitine palmitoyltransferase 1; PDK4, pyruvate dehydrogenase kinase-4; PGAM2, phosphoglycerate mutase 2; PGC-1α, PPAR coactivator 1α; PPAR, peroxisome proliferator-activated receptor; TA, tibial anterior; TWEAK, TNF-like weak inducer of apoptosis.

To understand the mechanisms by which TWEAK affects exercise capacity and mitochondrial content in skeletal muscle, we investigated whether TWEAK modulates the expression of PGC-1α, a critical regulator of the mitochondrial biogenetic program in skeletal muscle [9,10]. Interestingly, the mRNA levels of PGC-1α were found to be significantly higher in 4.5-month-old TWEAK-KO mice compared to age-matched wild-type mice (Figure 4). Similarly, mRNA levels of PPARδ were significantly higher in skeletal muscle of TWEAK-KO mice compared to corresponding wild-type mice (Figure 4). In contrast, mRNA levels of glycolytic enzymes such as hexokinase-2 (HK II) and phosphoglycerate mutase 2 (PGAM2) were reduced in skeletal muscle of TWEAK-KO mice compared to wild-type mice. No significant change was observed in the expression of pyruvate dehydrogenase kinase-4 (PDK4). Furthermore, mRNA levels of mitochondrial carnitine palmitoyltransferase I (mCPT1), which is required for the transport of long-chain fatty acyl-CoAs from the cytoplasm into the mitochondrion, were significantly elevated in TWEAK-KO mice compared to wild-type mice (Figure 4). These results suggest that a deficiency of TWEAK reduces expression of glycolytic genes and augments the expression of genes involved in oxidative metabolism in skeletal muscle.

### Ablation of TWEAK increases state 3 respiration in skeletal muscle mitochondria

**Figure 5:**
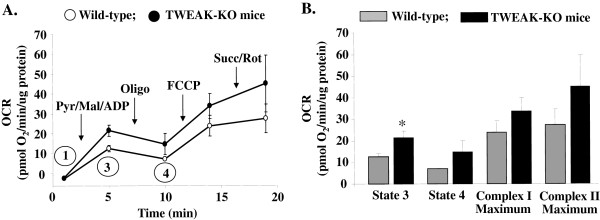
**Mitochondrial respiration is enhanced in TWEAK-KO mice.** Respiratory activity of mitochondria isolated from wild-type and TWEAK-KO mice subjected to an acute bout of treadmill running: **(A)** Extracellular flux assay of isolated mitochondria: mitochondrial activity was measured in the absence of substrate (state 1 respiration) or in the presence of pyruvate (5 mM), malate (2.5 mM) and ADP (1 mM) (state 3 respiration). State 4 respiration was estimated by measuring the oxygen consumption rate (OCR) after the addition of oligomycin (1 μg/ml). FCCP (4 μM) was added to assess maximal activity of mitochondria respiring on substrates providing electrons for complex I (that is, pyruvate and malate) or complex II (that is, succinate in the presence of rotenone; 10 mM and 1 μM, respectively). **(B)** Group data derived from panel A. N = 3 mice per group. *P <0.05 versus wild-type. FCCP, carbonyl cyanide *p*-trifluoromethoxyphenylhydrazone; KO, knockout; TWEAK, TNF-like weak inducer of apoptosis.

We next sought to determine whether TWEAK regulates mitochondrial function in skeletal muscle. After an acute bout of treadmill running for 90 minutes, mitochondria were isolated from hind limb muscles of wild type and TWEAK-KO mice and mitochondrial function was examined by extracellular flux (XF) analysis. The sequential addition of respiratory substrates and inhibitors of oxidative phosphorylation were used to determine changes in electron transport chain activity: state 3 respiration was induced by addition of pyruvate, malate and ADP; state 4 respiration was induced by addition of oligomycin; FCCP was added to determine maximal complex I-mediated respiratory activity; and, lastly, succinate and rotenone were added to determine maximal complex II-mediated respiratory activity. As shown in Figure 5A and 5B, mitochondria from TWEAK-KO mice showed significantly increased state 3 respiratory activities compared with wild type mice. Mitochondrial coupling (as determined by calculating respiratory control ratios) and other indices of mitochondrial function were not significantly different between wild type and KO mice. These data suggest that absence of TWEAK augments mitochondrial oxidative capacity in skeletal muscle in exercised mice.

### TWEAK represses the expression of PGC-1α and genes regulating oxidative metabolism in cultured primary myotubes

**Figure 6:**
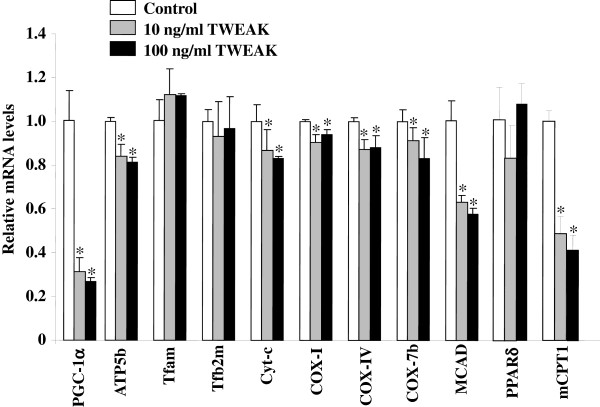
**Effect of TWEAK on expression of PGC-1α and mitochondria oxidative metabolism genes in cultured primary myotubes.** Primary myotubes prepared from wild-type mice were treated with soluble TWEAK protein (10 ng/ml or 100 ng/ml) for 72 hours followed by RNA isolation and performing qRT-PCR to study transcript levels of PGC-1α, Tfam, Tfam2m, Cyt-c, Cox I, Cox IV, Cox 7b, and MCAD. Data are presented as mean ± SD. *P <0.05; values vary significantly from untreated myotubes. Cox, cytochroms c oxidase; Cyt c, cytochrome c; MCAD, medium-chain acyl-coenzyme A dehydrogenase; PGC-1α, PPAR coactivator 1α; PPAR, peroxisome proliferator-activated receptor; Tfam, transcription factor A mitochondrial; TWEAK, TNF-like weak inducer of apoptosis.

We next studied whether TWEAK affects the expression of PGC-1α and other genes in cultured primary myotubes. Primary myoblasts prepared from wild-type mice were differentiated into myotubes by incubation in differentiation medium for 96 hours followed by treatment with TWEAK for 72 hours. As shown in Figure 6, addition of soluble TWEAK dramatically reduced the expression of PGC-1α in myotubes. Treatment with TWEAK also significantly reduced the mRNA levels of several mitochondrial genes encoding proteins related to oxidative metabolism, such as ATP synthase subunit beta (ATP5b), cytochrome c oxidase subunit I (Cox I), Cox subunit IV (COX IV), COX -7b, cytochrome c (Cyt c), medium-chain acyl-coenzyme A dehydrogenase (MCAD), PPARδ, and mCPT1 (Figure 6). These results further suggest that TWEAK inhibits the mitochondrial biogenetic program in skeletal muscle.

### TWEAK decreases maximal respiratory capacity and increases glycolysis in cultured primary myotubes

**Figure 7:**
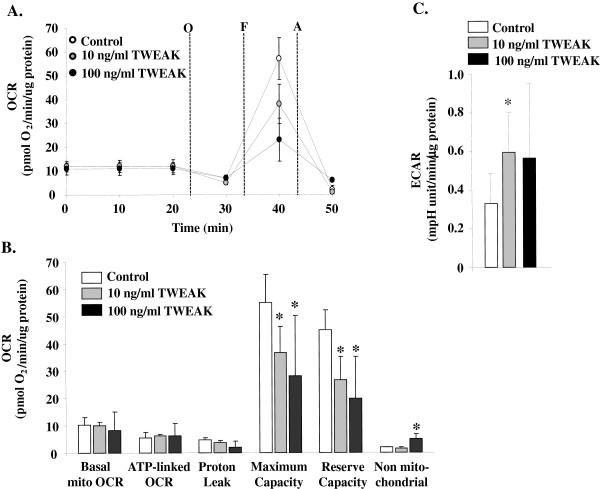
**TWEAK regulates mitochondrial oxidative capacity and glycolytic flux.** Extracellular flux analysis of primary differentiated myotubes treated with 0, 10, or 100 ng/ml TWEAK for 72 hours. **(A)** Mitochondrial function assay: after three baseline measurements, inhibitors or activators of electron transport were added sequentially to intact myotubes. Oxygen consumption rate (OCR) measurements were recorded after each exposure. O, oligomycin; F, FCCP; and A, antimycin A/rotenone. The OCR was normalized to total protein in each well. **(B)** Indices of mitochondrial function calculated from assay results in panel A. **(C)** Extracellular acidification rates (ECAR) of cells treated without or with TWEAK; ECAR is a surrogate measure of lactate and therefore is used as a measure of glycolytic flux. N = 3 per group; *P <0.05 versus control. FCCP, carbonyl cyanide *p*-trifluoromethoxyphenylhydrazone; TWEAK, TNF-like weak inducer of apoptosis.

Our studies in isolated mitochondria derived from skeletal muscle after exercise suggest that the absence of TWEAK increases mitochondrial oxidative capacity. To assess the effects of TWEAK on mitochondrial function directly, we exposed differentiated myotubes to TWEAK and then assessed mitochondrial function as described previously [48-51]. As shown in Figure 7A and 7B, exposure of myotubes to TWEAK for 72 hours did not affect the basal mitochondrial OCR, nor did it affect ATP-linked OCR or proton leak. However, the FCCP-stimulated OCR, indicative of maximal respiratory capacity, was remarkably diminished by TWEAK treatment. In addition, a modest, yet significant, increase in the non-mitochondrial OCR occurred in myotubes exposed to100 ng/ml TWEAK, which may be due to increased production of reactive oxygen species. The diminishment in mitochondrial respiratory capacity caused by TWEAK was associated with an increase in the extracellular acidification rate (ECAR), a surrogate measure of glycolysis (Figure 7C). Collectively, these results suggest that TWEAK directly modulates the bioenergetic capacity of skeletal muscle.

### Ablation of TWEAK improves angiogenesis in skeletal muscle of mice

**Figure 8:**
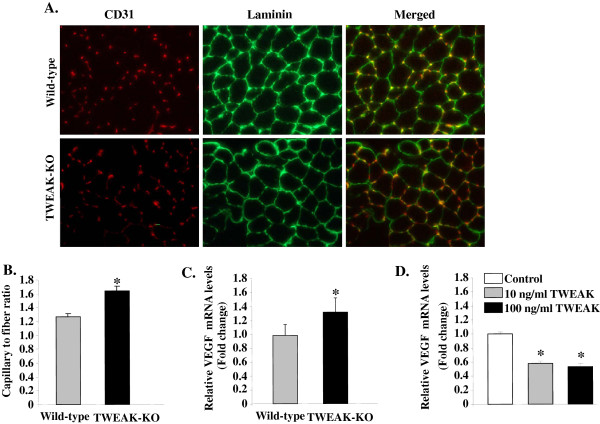
**Analysis of capillary density in skeletal muscle of wild-type and TWEAK-KO mice.** Transverse sections of TA muscle prepared from 4.5-month-old wild-type and TWEAK-KO mice were immunostained for CD31 (red) and counterstained for laminin (green). **(A)** Representative CD31- and laminin-immunostained and merged images are presented here. Scale bars: 50 μm. **(B)** Quantification of CD31-postive capillaries per myofiber in TA muscle. N = 8 in each group. **(C)** Relative mRNA levels of VEGF in TA muscle of wild-type and TWEAK-KO mice (N = 4 in each group) measured by qRT-PCR analysis. **(D)** Primary myotubes were treated with the indicated concentration of TWEAK for 24 hours followed by measurement of mRNA levels of VEGF by qRT-PCR. Relative mRNA levels of VEGF are shown here. Data are presented as mean ± SD. *P <0.05; values vary significantly from untreated myotubes. KO, knockout; TA, tibial anterior; TWEAK, TNF-like weak inducer of apoptosis; VEGF, vascular endothelial growth factor.

There is a causal relationship between PGC-1α and angiogenesis. Skeletal muscle-specific overexpression of PGC-1α in mice results in increased angiogenesis, which may contribute to enhanced exercise performance [52]. Since TWEAK suppresses PGC-α in skeletal muscle, we next sought to determine whether TWEAK also regulates angiogenesis in skeletal muscle of mice. TA muscle of 4.5-month-old wild-type and TWEAK-KO mice was isolated and immunostained with antibodies against CD31 (also known as platelet endothelial cell adhesion molecule-1), an endothelial-specific marker for capillaries. Laminin staining was used to mark the periphery of fibers. As shown in Figure 8A, the number of CD31-positive capillaries was considerably increased in TWEAK-KO mice compared with wild-type mice. The capillary-to-fiber ratio was increased by 29% in TWEAK-KO mice (Figure 8B). Since VEGF positively regulates angiogenesis, we also measured mRNA levels of VEGF in TA muscle of wild-type and TWEAK-KO mice. As shown in Figure 8C, mRNA levels of VEGF were significantly higher in TA muscle of TWEAK-KO mice compared to wild-type mice. Furthermore, treatment with TWEAK also reduced the mRNA levels of VEGF in primary myotubes (Figure 8D). These results suggest that ablation of TWEAK stimulates angiogenesis in skeletal muscle of mice potentially through increasing the expression of VEGF.

## Discussion

Exercise capacity in mammals is determined by multiple factors including skeletal muscle oxidative metabolism and vascularization. Our previous microarray study suggested that TWEAK can modulate the expression of several genes whose products are involved in mitochondrial dysfunction and fatty acid metabolism [53]. Our present study demonstrates that compared to wild-type mice, TWEAK-KO mice run longer and with higher speed during an exercise tolerance test (Figure 1). TWEAK-KO mice show augmented levels of subsarcolemmal and intermyofibrillar mitochondria, increased SDH-positive myofibers, and elevated expression of metabolic genes such as PGC-1α, PPARδ, and mCPT-1 compared to wild-type mice (Figures 2, 3, and 4). Oxidative phosphorylation is also increased in exercised TWEAK-KO mice compared with wild-type mice (Figure 5), and treatment of myotubes with TWEAK directly decreases mitochondrial biogenetic capacity and maximal respiratory activity (Figures 6 and 7). Moreover, vascularization and expression of VEGF in skeletal muscle are enhanced in TWEAK-KO mice compared to their controls (Figure 8). These data suggest that TWEAK directly regulates the mitochondrial biogenetic program and that loss of TWEAK improves mitochondrial respiratory capacity and increases vascularization of skeletal muscle tissue, which collectively lead to improved exercise performance.

One of the potential mechanisms by which TWEAK might be attenuating exercise capacity is through diminishing the levels of PGC-1α in skeletal muscle. PGC-1α augments mitochondrial biogenesis, oxidative metabolism, and promotes fast-to-slow type fiber transition [9,13,17,54-56]. Overexpression of PGC-1α in rat primary culture cells leads to increased abundance of slow oxidative-associated MyHC isoform [57]. Skeletal muscle-specific PGC-1α KO mice demonstrate reduced endurance capacity with a shift from oxidative fiber type to glycolytic fibers and increased levels of TNF-α in skeletal muscle [45,58]. By contrast, muscle-specific overexpression of PGC-1α enhances exercise performance with increased fatty acid oxidation and decreased glycogen usage during exercise [8,52]. These findings suggest that PGC-1α is not only essential but sufficient to determining skeletal muscle fiber composition. Our experiments demonstrate that mRNA levels of PGC-1α are increased in skeletal muscle of TWEAK-KO mice with a concomitant increase in mitochondrial content and expression of genes whose products are involved in oxidative capacity (Figures 2 and 4). Furthermore, treatment of primary myotubes with TWEAK drastically reduced levels of PGC-1α and other mitochondrial genes (Figure 6) further suggesting that TWEAK represses the expression of PGC-1α leading to reduced mitochondrial content. These findings are consistent with functional analyses of mitochondrial function: ablation of TWEAK resulted in increased oxidative phosphorylation capacity in isolated mitochondria (Figure 5) and treatment of myotubes with TWEAK decreased maximal respiratory capacity (Figure 7). Although not tested in this study, it is possible that TWEAK also reduces the levels of PGC-1α in other organs which ultimately results in reduced exercise capacity. Indeed, Shi *et al*. recently reported that TWEAK represses the expression of PGC-1α and other molecules involved in mitochondrial oxidative phosphorylation in cardiomyocytes and forced expression of PGC-1α attenuates TWEAK-induced cardiac dysfunction in mice [59].

The PPARs are ligand-modulated transcription factors in which three subtypes have been identified: α, β/δ, and γ [60]. Previous studies have shown that their endogenous ligands are composed of fatty acids and lipid metabolites and, therefore, certain PPARs mediate the expression of genes whose products are involved in the regulation of fatty acid metabolism in response to changes in systemic fuel availability [60-62]. In skeletal muscle, levels of PPARδ are relatively higher compared to PPARα or PPARγ [63]. Treatment with synthetic PPARδ agonist or overexpression of PPARδ by retroviral infection induces the levels of molecules which are involved in lipid metabolism and fatty acid oxidation, whereas overexpression of a dominant-negative PPARδ mutant exerts opposite effects in C2C12 myotubes [64]. Furthermore, it has been shown that muscle-specific overexpression of PPARδ in mice increases exercise tolerance with a switch to increased number of type I muscle fibers and up-regulation of molecules related to fatty acid metabolism. In contrast, PPARδ-null mice show decreased exercise performance compared to wild-type mice [65,66]. Although muscle relies mainly on fat and carbohydrate as energy resources, enhanced fatty acid utilization during exercise with glycogen sparing results in improved exercise endurance capacity [67-69]. Haramizu *et al*. demonstrated that the expression levels of gene products related to lipid metabolism in the muscle is correlated with the levels of fatty acid β-oxidation activity as well as exercise strength [70]. Our results demonstrate that transcript levels of PPARδ and mCPT1 are significantly increased while the expression of molecules that are associated with glycolysis such as HK II and PGAM2 is suppressed in skeletal muscle of TWEAK-KO mice compared to wild-type mice (Figure 6). These results suggest that enhanced fatty acid oxidation might be responsible for improvement in exercise capacity in TWEAK-KO mice. Data from cell culture experiments further support this possibility, as treatment of myotubes with TWEAK resulted in diminished maximal respiratory capacity and increased glycolytic flux (Figure 7).

Although the mechanisms by which TWEAK reduces levels of PGC-1α in skeletal muscle remain unknown, a recent study has shown that TWEAK increases membrane translocation of adaptor protein TNF receptor associated factor 2 (TRAF2), in an Fn14 dependent manner, in cardiomyocytes [59]. Furthermore, TWEAK treatment increases the activation of canonical nuclear factor-kappaB (NF-κB) signaling in both skeletal muscle [41] and cardiac myocytes [59]. Knockdown of TRAF2 using small hairpin RNA (shRNA) or selective blockade of IκB kinase-β (IKKβ, an upstream activator of canonical NF-κB signaling) prevented the TWEAK-mediated suppression of PGC-1α in cardiomyocytes suggesting that TWEAK repression of PGC-1α requires Fn14-TRAF2-IKKβ-NF-κB signaling cascade [59]. Previously, the effects of TNF-α on the mRNA expression of PPARδ and its target genes have been investigated. Treatment with TNF-α reduces PPARδ-target genes, such as mCPT1 and PGC-1α, whereas addition of PPARδ agonist rescues this reduction in adipocytes [71,72]. Moreover, TNF-α impairs mitochondrial biogenesis and function in skeletal muscle [73]. While TWEAK can induce the expression of molecules associated with the autophagy-lysosomal system indicating it causes mitochondria dysfunction [74], muscle-specific ablation of TRAF6, which is involved in TWEAK signaling, suppresses the activation of autophagy in response to denervation and cancer cachexia [75]. These results suggest that TWEAK is involved in reducing PPARδ and PGC-1α levels and their target genes leading to mitochondrial dysfunction and content. Since no change of PPARδ expression was observed in skeletal muscle of PGC-1α KO mice or the muscle-specific PGC-1α overexpressing transgenic mice [66], PGC-1α is not an upstream regulator for PPARδ. Indeed, PGC-1α has a synergistic effect with PPARδ agonist to induce the expression of oxidative metabolic genes such as *mCPT1* and *PDK4*[76]. Furthermore, it has been shown that PGC-1α directly coactivates the *mCPT1* and *PDK4* promoter via PPARδ in a ligand-dependent manner [76,77]. Therefore, it is likely that PGC-1α is a coactivator of PPARδ in terms of up-regulating these metabolic genes.

Another interesting observation of the present study is that depletion of TWEAK improves angiogenesis in skeletal muscle of mice (Figure 8). Previous studies have shown that transgenic mice overexpressing PGC-1α show increased vascularization in skeletal muscle [56]. PGC-1α is also a positive regulator for the expression of VEGF1 which is known to promote angiogenesis [56]. Moreover, human endothelial cells treated with the PPARδ agonist GW501516 demonstrated increased production of VEGF and expression of VEGF receptor [78,79]. Therefore, increased angiogenesis in skeletal muscle of TWEAK-KO mice appears to be a result of increased amounts of PGC-1α and PPAR δ (Figure 4). The enhancement of vascularization in TWEAK-KO mice might further augment the capacity of skeletal muscle mitochondria to respire by increasing blood flow and delivery of O_2_ and nutrients, especially during exercise.

## Conclusion

In summary, our study provides initial evidence that inhibition of TWEAK increases mitochondrial biogenesis, oxidative metabolism and angiogenesis in skeletal muscle which potentially contribute to improved exercise capacity. These findings also have high clinical significance as inhibition of TWEAK activity using neutralizing antibodies or pharmacological compounds could improve muscle function and exercise capacity in patients with metabolic disorders.

## Abbreviations

ATP5b: ATP synthase subunit beta; BSA: Bovine serum albumin; Cox: Cytochrome c oxidase; FCCP: Carbonyl cyanide *p*-trifluoromethoxyphenylhydrazone; GA: Gastrocnemius; HK II: Hexokinase II; KO: Knockout; MCAD: Medium-chain acyl-coenzyme A dehydrogenase; mCPT1: Mitochondrial carnitine palmitoyltransferase I; MyHC: Myosin heavy chain; NF-κB: Nuclear factor-kappaB; OCR: Oxygen consumption rate; PCR: Polymerase chain reaction; PDK4: Pyruvate dehydrogenase kinase 4; PGAM2: Phosphoglycerate mutase 2; PGC-1α: PPAR coactivator 1α; PPAR: Peroxisome proliferator-activated receptor; qRT-PCR: Quantitative real-time PCR; SDH: Succinate dehydrogenase; shRNA: Small hairpin RNA; TA: Tibial anterior; TNF: Tumor necrosis factor; TRAF: TNF receptor-associated factor; TWEAK: TNF-like weak inducer of apoptosis; VEGF: Vascular endothelial growth factor.

## Competing interests

The authors declare they have no competing interests.

## Authors’ contributions

AK and BGH conceived and designed the study. SS, YO, VM, JS, BGH and SB performed experiments and analyzed the data. SS, BGH and AK wrote the manuscript. All authors read and approved the final manuscript.

## References

[B1] Pette D The adaptive potential of skeletal muscle fibers Can J Appl Physiol 2002 27 423 448 10.1139/h02-023 12442355

[B2] Holloszy JO Biochemical adaptations in muscle. Effects of exercise on mitochondrial oxygen uptake and respiratory enzyme activity in skeletal muscle J Biol Chem 1967 242 2278 2282 4290225

[B3] Lanza IR Sreekumaran Nair K Regulation of skeletal muscle mitochondrial function: genes to proteins Acta Physiol (Oxf) 2010 199 529 547 10.1111/j.1748-1716.2010.02124.x 20345409 PMC3070482

[B4] Arany Z PGC-1 coactivators and skeletal muscle adaptations in health and disease Curr Opin Genet Dev 2008 18 426 434 10.1016/j.gde.2008.07.018 18782618 PMC2629557

[B5] Finck BN Kelly DP PGC-1 coactivators: inducible regulators of energy metabolism in health and disease J Clin Invest 2006 116 615 622 10.1172/JCI27794 16511594 PMC1386111

[B6] Uldry M Yang W St-Pierre J Lin J Seale P Spiegelman BM Complementary action of the PGC-1 coactivators in mitochondrial biogenesis and brown fat differentiation Cell Metab 2006 3 333 341 10.1016/j.cmet.2006.04.002 16679291

[B7] Arany Z Lebrasseur N Morris C Smith E Yang W Ma Y Chin S Spiegelman BM The transcriptional coactivator PGC-1beta drives the formation of oxidative type IIX fibers in skeletal muscle Cell Metab 2007 5 35 46 10.1016/j.cmet.2006.12.003 17189205

[B8] Calvo JA Daniels TG Wang X Paul A Lin J Spiegelman BM Stevenson SC Rangwala SM Muscle-specific expression of PPARgamma coactivator-1alpha improves exercise performance and increases peak oxygen uptake J Appl Physiol 2008 104 1304 1312 10.1152/japplphysiol.01231.2007 18239076

[B9] Lin J Wu H Tarr PT Zhang CY Wu Z Boss O Michael LF Puigserver P Isotani E Olson EN Lowell BB Bassel-Duby R Spiegelman BM Transcriptional co-activator PGC-1 alpha drives the formation of slow-twitch muscle fibres Nature 2002 418 797 801 10.1038/nature00904 12181572

[B10] Wende AR Schaeffer PJ Parker GJ Zechner C Han DH Chen MM Hancock CR Lehman JJ Huss JM McClain DA Holloszy JO Kelly DP A role for the transcriptional coactivator PGC-1alpha in muscle refueling J Biol Chem 2007 282 36642 36651 10.1074/jbc.M707006200 17932032

[B11] Sandri M Lin J Handschin C Yang W Arany ZP Lecker SH Goldberg AL Spiegelman BM PGC-1alpha protects skeletal muscle from atrophy by suppressing FoxO3 action and atrophy-specific gene transcription Proc Natl Acad Sci U S A 2006 103 16260 16265 10.1073/pnas.0607795103 17053067 PMC1637570

[B12] Brault JJ Jespersen JG Goldberg AL Peroxisome proliferator-activated receptor gamma coactivator 1alpha or 1beta overexpression inhibits muscle protein degradation, induction of ubiquitin ligases, and disuse atrophy J Biol Chem 2010 285 19460 19471 10.1074/jbc.M110.113092 20404331 PMC2885225

[B13] Wu Z Puigserver P Andersson U Zhang C Adelmant G Mootha V Troy A Cinti S Lowell B Scarpulla RC Spiegelman BM Mechanisms controlling mitochondrial biogenesis and respiration through the thermogenic coactivator PGC-1 Cell 1999 98 115 124 10.1016/S0092-8674(00)80611-X 10412986

[B14] Mootha VK Handschin C Arlow D Xie X St Pierre J Sihag S Yang W Altshuler D Puigserver P Patterson N Willy PJ Schulman IG Heyman RA Lander ES Spiegelman BM Erralpha and Gabpa/b specify PGC-1alpha-dependent oxidative phosphorylation gene expression that is altered in diabetic muscle Proc Natl Acad Sci USA 2004 101 6570 6575 10.1073/pnas.0401401101 15100410 PMC404086

[B15] Huss JM Kopp RP Kelly DP Peroxisome proliferator-activated receptor coactivator-1alpha (PGC-1alpha) coactivates the cardiac-enriched nuclear receptors estrogen-related receptor-alpha and -gamma. Identification of novel leucine-rich interaction motif within PGC-1alpha J Biol Chem 2002 277 40265 40274 10.1074/jbc.M206324200 12181319

[B16] Huss JM Torra IP Staels B Giguere V Kelly DP Estrogen-related receptor alpha directs peroxisome proliferator-activated receptor alpha signaling in the transcriptional control of energy metabolism in cardiac and skeletal muscle Mol Cell Biol 2004 24 9079 9091 10.1128/MCB.24.20.9079-9091.2004 15456881 PMC517878

[B17] Puigserver P Wu Z Park CW Graves R Wright M Spiegelman BM A cold-inducible coactivator of nuclear receptors linked to adaptive thermogenesis Cell 1998 92 829 839 10.1016/S0092-8674(00)81410-5 9529258

[B18] Scarpulla RC Transcriptional paradigms in mammalian mitochondrial biogenesis and function Physiol Rev 2008 88 611 638 10.1152/physrev.00025.2007 18391175

[B19] Romanello V Guadagnin E Gomes L Roder I Sandri C Petersen Y Milan G Masiero E Del Piccolo P Foretz M Scorrano L Rudolf R Sandri M Mitochondrial fission and remodelling contributes to muscle atrophy EMBO J 2010 29 1774 1785 10.1038/emboj.2010.60 20400940 PMC2876965

[B20] Lokireddy S Wijesoma IW Teng S Bonala S Gluckman PD McFarlane C Sharma M Kambadur R The ubiquitin ligase Mul1 induces mitophagy in skeletal muscle in response to muscle-wasting stimuli Cell Metab 2012 16 613 624 10.1016/j.cmet.2012.10.005 23140641

[B21] Argiles JM Busquets S Toledo M Lopez-Soriano FJ The role of cytokines in cancer cachexia Curr Opin Support Palliat Care 2009 3 263 268 10.1097/SPC.0b013e3283311d09 19713854

[B22] Carson JA Baltgalvis KA Interleukin 6 as a key regulator of muscle mass during cachexia Exerc Sport Sci Rev 2010 38 168 176 10.1097/JES.0b013e3181f44f11 20871233 PMC3065300

[B23] Hardin BJ Campbell KS Smith JD Arbogast S Smith J Moylan JS Reid MB TNF-alpha acts via TNFR1 and muscle-derived oxidants to depress myofibrillar force in murine skeletal muscle J Appl Physiol 2008 104 694 699 10.1152/japplphysiol.00898.2007 18187611

[B24] Reid MB Lannergren J Westerblad H Respiratory and limb muscle weakness induced by tumor necrosis factor-alpha: involvement of muscle myofilaments Am J Respir Crit Care Med 2002 166 479 484 10.1164/rccm.2202005 12186824

[B25] Li YP Schwartz RJ Waddell ID Holloway BR Reid MB Skeletal muscle myocytes undergo protein loss and reactive oxygen-mediated NF-kappaB activation in response to tumor necrosis factor alpha FASEB J 1998 12 871 880 9657527 10.1096/fasebj.12.10.971

[B26] Li YP Reid MB NF-kappaB mediates the protein loss induced by TNF-alpha in differentiated skeletal muscle myotubes Am J Physiol Regul Integr Comp Physiol 2000 279 R1165 R1170 11003979 10.1152/ajpregu.2000.279.4.R1165

[B27] Reid MB Moylan JS Beyond atrophy: redox mechanisms of muscle dysfunction in chronic inflammatory disease J Physiol 2011 589 2171 2179 10.1113/jphysiol.2010.203356 21320886 PMC3098696

[B28] Romanatto T Roman EA Arruda AP Denis RG Solon C Milanski M Moraes JC Bonfleur ML Degasperi GR Picardi PK Hirabara S Boschero AC Curi R Velloso LA Deletion of tumor necrosis factor-alpha receptor 1 (TNFR1) protects against diet-induced obesity by means of increased thermogenesis J Biol Chem 2009 284 36213 36222 10.1074/jbc.M109.030874 19858212 PMC2794737

[B29] Tang K Wagner PD Breen EC TNF-alpha-mediated reduction in PGC-1alpha may impair skeletal muscle function after cigarette smoke exposure J Cell Physiol 2010 222 320 327 10.1002/jcp.21955 19859910 PMC5831677

[B30] White JP Puppa MJ Sato S Gao S Price RL Baynes JW Kostek MC Matesic LE Carson JA IL-6 regulation on skeletal muscle mitochondrial remodeling during cancer cachexia in the ApcMin/+ mouse Skelet Muscle 2012 2 14 10.1186/2044-5040-2-14 22769563 PMC3431229

[B31] White JP Baltgalvis KA Puppa MJ Sato S Baynes JW Carson JA Muscle oxidative capacity during IL-6-dependent cancer cachexia Am J Physiol Regul Integr Comp Physiol 2011 300 R201 R211 10.1152/ajpregu.00300.2010 21148472 PMC3043802

[B32] Mangner N Linke A Oberbach A Kullnick Y Gielen S Sandri M Hoellriegel R Matsumoto Y Schuler G Adams V Exercise training prevents TNF-alpha induced loss of force in the diaphragm of mice PLoS One 2013 8 e52274 10.1371/journal.pone.0052274 23300968 PMC3534708

[B33] Santos RV Viana VA Boscolo RA Marques VG Santana MG Lira FS Tufik S de Mello MT Moderate exercise training modulates cytokine profile and sleep in elderly people Cytokine 2012 60 731 735 10.1016/j.cyto.2012.07.028 22917967

[B34] Rubin DA Hackney AC Inflammatory cytokines and metabolic risk factors during growth and maturation: influence of physical activity Med Sport Sci 2010 55 43 55 20956859 10.1159/000321971

[B35] Christiansen T Bruun JM Paulsen SK Olholm J Overgaard K Pedersen SB Richelsen B Acute exercise increases circulating inflammatory markers in overweight and obese compared with lean subjects Eur J Appl Physiol 2013 113 1635 1642 10.1007/s00421-013-2592-0 23361845

[B36] Chicheportiche Y Bourdon PR Xu H Hsu YM Scott H Hession C Garcia I Browning JL TWEAK, a new secreted ligand in the tumor necrosis factor family that weakly induces apoptosis J Biol Chem 1997 272 32401 32410 10.1074/jbc.272.51.32401 9405449

[B37] Meighan-Mantha RL Hsu DK Guo Y Brown SA Feng SL Peifley KA Alberts GF Copeland NG Gilbert DJ Jenkins NA Richards CM Winkles JA The mitogen-inducible Fn14 gene encodes a type I transmembrane protein that modulates fibroblast adhesion and migration J Biol Chem 1999 274 33166 33176 10.1074/jbc.274.46.33166 10551889

[B38] Winkles JA Tran NL Brown SA Stains N Cunliffe HE Berens ME Role of TWEAK and Fn14 in tumor biology Front Biosci 2007 12 2761 2771 10.2741/2270 17127278

[B39] Wiley SR Cassiano L Lofton T Davis-Smith T Winkles JA Lindner V Liu H Daniel TO Smith CA Fanslow WC A novel TNF receptor family member binds TWEAK and is implicated in angiogenesis Immunity 2001 15 837 846 10.1016/S1074-7613(01)00232-1 11728344

[B40] Maecker H Varfolomeev E Kischkel F Lawrence D LeBlanc H Lee W Hurst S Danilenko D Li J Filvaroff E Yang B Daniel D Ashkenazi A TWEAK attenuates the transition from innate to adaptive immunity Cell 2005 123 931 944 10.1016/j.cell.2005.09.022 16325585

[B41] Mittal A Bhatnagar S Kumar A Lach-Trifilieff E Wauters S Li H Makonchuk DY Glass DJ Kumar A The TWEAK-Fn14 system is a critical regulator of denervation-induced skeletal muscle atrophy in mice J Cell Biol 2010 188 833 849 10.1083/jcb.200909117 20308426 PMC2845082

[B42] Kumar A Bhatnagar S Paul PK TWEAK and TRAF6 regulate skeletal muscle atrophy Curr Opin Clin Nutr Metab Care 2012 15 233 239 10.1097/MCO.0b013e328351c3fc 22366923 PMC3397822

[B43] Dahiya S Bhatnagar S Hindi SM Jiang C Paul PK Kuang S Kumar A Elevated levels of active matrix metalloproteinase-9 cause hypertrophy in skeletal muscle of normal and dystrophin-deficient mdx mice Hum Mol Genet 2011 20 4345 4359 10.1093/hmg/ddr362 21846793 PMC3196885

[B44] Safdar A Little JP Stokl AJ Hettinga BP Akhtar M Tarnopolsky MA Exercise increases mitochondrial PGC-1alpha content and promotes nuclear-mitochondrial cross-talk to coordinate mitochondrial biogenesis J Biol Chem 2011 286 10605 10617 10.1074/jbc.M110.211466 21245132 PMC3060512

[B45] Handschin C Chin S Li P Liu F Maratos-Flier E Lebrasseur NK Yan Z Spiegelman BM Skeletal muscle fiber-type switching, exercise intolerance, and myopathy in PGC-1alpha muscle-specific knock-out animals J Biol Chem 2007 282 30014 30021 10.1074/jbc.M704817200 17702743

[B46] Nachlas MM Tsou KC De Souza E Cheng CS Seligman AM Cytochemical demonstration of succinic dehydrogenase by the use of a new p-nitrophenyl substituted ditetrazole J Histochem Cytochem 1957 5 420 436 10.1177/5.4.420 13463314

[B47] Sauerbeck A Pandya J Singh I Bittman K Readnower R Bing G Sullivan P Analysis of regional brain mitochondrial bioenergetics and susceptibility to mitochondrial inhibition utilizing a microplate based system J Neurosci Methods 2011 198 36 43 10.1016/j.jneumeth.2011.03.007 21402103 PMC3535268

[B48] Hill BG Dranka BP Zou L Chatham JC Darley-Usmar VM Importance of the bioenergetic reserve capacity in response to cardiomyocyte stress induced by 4-hydroxynonenal Biochem J 2009 424 99 107 10.1042/BJ20090934 19740075 PMC2872628

[B49] Dranka BP Hill BG Darley-Usmar VM Mitochondrial reserve capacity in endothelial cells: the impact of nitric oxide and reactive oxygen species Free Radic Biol Med 2010 48 905 914 10.1016/j.freeradbiomed.2010.01.015 20093177 PMC2860730

[B50] Perez J Hill BG Benavides GA Dranka BP Darley-Usmar VM Role of cellular bioenergetics in smooth muscle cell proliferation induced by platelet-derived growth factor Biochem J 2010 428 255 267 10.1042/BJ20100090 20331438 PMC3641000

[B51] Hill BG Benavides GA Lancaster JR Ballinger S Dell’italia L Zhang J Darley-Usmar VM Integration of cellular bioenergetics with mitochondrial quality control and autophagy Biol Chem 2012 393 1485 1512 23092819 10.1515/hsz-2012-0198 PMC3594552

[B52] Tadaishi M Miura S Kai Y Kano Y Oishi Y Ezaki O Skeletal muscle-specific expression of PGC-1alpha-b, an exercise-responsive isoform, increases exercise capacity and peak oxygen uptake PLoS One 2011 6 e28290 10.1371/journal.pone.0028290 22174785 PMC3234261

[B53] Panguluri SK Bhatnagar S Kumar A McCarthy JJ Srivastava AK Cooper NG Lundy RF Genomic profiling of messenger RNAs and microRNAs reveals potential mechanisms of TWEAK-induced skeletal muscle wasting in mice PLoS One 2010 5 e8760 10.1371/journal.pone.0008760 20098732 PMC2808241

[B54] Vega RB Huss JM Kelly DP The coactivator PGC-1 cooperates with peroxisome proliferator-activated receptor alpha in transcriptional control of nuclear genes encoding mitochondrial fatty acid oxidation enzymes Mol Cell Biol 2000 20 1868 1876 10.1128/MCB.20.5.1868-1876.2000 10669761 PMC85369

[B55] Liang H Ward WF PGC-1alpha: a key regulator of energy metabolism Adv Physiol Educ 2006 30 145 151 10.1152/advan.00052.2006 17108241

[B56] Chinsomboon J Ruas J Gupta RK Thom R Shoag J Rowe GC Sawada N Raghuram S Arany Z The transcriptional coactivator PGC-1alpha mediates exercise-induced angiogenesis in skeletal muscle Proc Natl Acad Sci U S A 2009 106 21401 21406 10.1073/pnas.0909131106 19966219 PMC2795492

[B57] Mortensen OH Frandsen L Schjerling P Nishimura E Grunnet N PGC-1alpha and PGC-1beta have both similar and distinct effects on myofiber switching toward an oxidative phenotype Am J Physiol Endocrinol Metab 2006 291 E807 E816 10.1152/ajpendo.00591.2005 16720625

[B58] Leone TC Lehman JJ Finck BN Schaeffer PJ Wende AR Boudina S Courtois M Wozniak DF Sambandam N Bernal-Mizrachi C Chen Z Holloszy JO Medeiros DM Schmidt RE Saffitz JE Abel ED Semenkovich CF Kelly DP PGC-1alpha deficiency causes multi-system energy metabolic derangements: muscle dysfunction, abnormal weight control and hepatic steatosis PLoS Biol 2005 3 e101 10.1371/journal.pbio.0030101 15760270 PMC1064854

[B59] Shi J Jiang B Qiu Y Guan J Jain M Cao X Bauer M Su L Burkly LC Leone TC Kelly DP Liao R PGC1alpha plays a critical role in TWEAK-induced cardiac dysfunction PLoS One 2013 8 e54054 10.1371/journal.pone.0054054 23342071 PMC3546975

[B60] Kliewer SA Xu HE Lambert MH Willson TM Peroxisome proliferator-activated receptors: from genes to physiology Recent Prog Horm Res 2001 56 239 263 10.1210/rp.56.1.239 11237216

[B61] Takahashi S Tanaka T Sakai J New therapeutic target for metabolic syndrome: PPARdelta Endocr J 2007 54 347 357 10.1507/endocrj.KR-99 17409576

[B62] Fredenrich A Grimaldi PA PPAR delta: an uncompletely known nuclear receptor Diabetes Metab 2005 31 23 27 10.1016/S1262-3636(07)70162-3 15803109

[B63] Muoio DM MacLean PS Lang DB Li S Houmard JA Way JM Winegar DA Corton JC Dohm GL Kraus WE Fatty acid homeostasis and induction of lipid regulatory genes in skeletal muscles of peroxisome proliferator-activated receptor (PPAR) alpha knock-out mice. Evidence for compensatory regulation by PPAR delta J Biol Chem 2002 277 26089 26097 10.1074/jbc.M203997200 12118038

[B64] Holst D Luquet S Nogueira V Kristiansen K Leverve X Grimaldi PA Nutritional regulation and role of peroxisome proliferator-activated receptor delta in fatty acid catabolism in skeletal muscle Biochim Biophys Acta 2003 1633 43 50 10.1016/S1388-1981(03)00071-4 12842194

[B65] Luquet S Lopez-Soriano J Holst D Fredenrich A Melki J Rassoulzadegan M Grimaldi PA Peroxisome proliferator-activated receptor delta controls muscle development and oxidative capability FASEB J 2003 17 2299 2301 14525942 10.1096/fj.03-0269fje

[B66] Wang YX Zhang CL Yu RT Cho HK Nelson MC Bayuga-Ocampo CR Ham J Kang H Evans RM Regulation of muscle fiber type and running endurance by PPARdelta PLoS Biol 2004 2 e294 10.1371/journal.pbio.0020294 15328533 PMC509410

[B67] Hawley JA Brouns F Jeukendrup A Strategies to enhance fat utilisation during exercise Sports Med 1998 25 241 257 10.2165/00007256-199825040-00003 9587182

[B68] Helge JW Long-term fat diet adaptation effects on performance, training capacity, and fat utilization Med Sci Sports Exerc 2002 34 1499 1504 10.1097/00005768-200209000-00016 12218745

[B69] Miller WC Bryce GR Conlee RK Adaptations to a high-fat diet that increase exercise endurance in male rats J Appl Physiol 1984 56 78 83 6693336 10.1152/jappl.1984.56.1.78

[B70] Haramizu S Nagasawa A Ota N Hase T Tokimitsu I Murase T Different contribution of muscle and liver lipid metabolism to endurance capacity and obesity susceptibility of mice J Appl Physiol 2009 106 871 879 10.1152/japplphysiol.90804.2008 19131482

[B71] Rodriguez-Calvo R Serrano L Coll T Moullan N Sanchez RM Merlos M Palomer X Laguna JC Michalik L Wahli W Vazquez-Carrera M Activation of peroxisome proliferator-activated receptor beta/delta inhibits lipopolysaccharide-induced cytokine production in adipocytes by lowering nuclear factor-kappaB activity via extracellular signal-related kinase 1/2 Diabetes 2008 57 2149 2157 10.2337/db08-0176 18443198 PMC2494695

[B72] Serrano-Marco L Chacon MR Maymo-Masip E Barroso E Salvado L Wabitsch M Garrido-Sanchez L Tinahones FJ Palomer X Vendrell J Vazquez-Carrera M TNF-alpha inhibits PPARbeta/delta activity and SIRT1 expression through NF-kappaB in human adipocytes Biochim Biophys Acta 2012 1821 1177 1185 10.1016/j.bbalip.2012.05.006 22683888

[B73] Valerio A Cardile A Cozzi V Bracale R Tedesco L Pisconti A Palomba L Cantoni O Clementi E Moncada S Carruba MO Nisoli E TNF-alpha downregulates eNOS expression and mitochondrial biogenesis in fat and muscle of obese rodents J Clin Invest 2006 116 2791 2798 16981010 10.1172/JCI28570 PMC1564431

[B74] Bhatnagar S Mittal A Gupta SK Kumar A TWEAK causes myotube atrophy through coordinated activation of ubiquitin-proteasome system, autophagy, and caspases J Cell Physiol 2012 227 1042 1051 10.1002/jcp.22821 21567392 PMC4154369

[B75] Paul PK Gupta SK Bhatnagar S Panguluri SK Darnay BG Choi Y Kumar A Targeted ablation of TRAF6 inhibits skeletal muscle wasting in mice J Cell Biol 2010 191 1395 1411 10.1083/jcb.201006098 21187332 PMC3010064

[B76] Kleiner S Nguyen-Tran V Bare O Huang X Spiegelman B Wu Z PPAR{delta} agonism activates fatty acid oxidation via PGC-1{alpha} but does not increase mitochondrial gene expression and function J Biol Chem 2009 284 18624 18633 10.1074/jbc.M109.008797 19435887 PMC2707195

[B77] Dressel U Allen TL Pippal JB Rohde PR Lau P Muscat GE The peroxisome proliferator-activated receptor beta/delta agonist, GW501516, regulates the expression of genes involved in lipid catabolism and energy uncoupling in skeletal muscle cells Mol Endocrinol 2003 17 2477 2493 10.1210/me.2003-0151 14525954

[B78] Piqueras L Reynolds AR Hodivala-Dilke KM Alfranca A Redondo JM Hatae T Tanabe T Warner TD Bishop-Bailey D Activation of PPARbeta/delta induces endothelial cell proliferation and angiogenesis Arterioscler Thromb Vasc Biol 2007 27 63 69 10.1161/01.ATV.0000250972.83623.61 17068288

[B79] Stephen RL Gustafsson MC Jarvis M Tatoud R Marshall BR Knight D Ehrenborg E Harris AL Wolf CR Palmer CN Activation of peroxisome proliferator-activated receptor delta stimulates the proliferation of human breast and prostate cancer cell lines Cancer Res 2004 64 3162 3170 10.1158/0008-5472.CAN-03-2760 15126355

